# An Optical Sensor for Dengue Envelope Proteins Using Polyamidoamine Dendrimer Biopolymer-Based Nanocomposite Thin Film: Enhanced Sensitivity, Selectivity, and Recovery Studies

**DOI:** 10.3390/polym13050762

**Published:** 2021-02-28

**Authors:** Nur Alia Sheh Omar, Yap Wing Fen, Irmawati Ramli, Amir Reza Sadrolhosseini, Jaafar Abdullah, Nor Azah Yusof, Yasmin Mustapha Kamil, Mohd Adzir Mahdi

**Affiliations:** 1 Faculty of Science, Universiti Putra Malaysia, UPM Serdang 43400, Selangor, Malaysia; nuralia.upm@gmail.com (N.A.S.O.); irmawati@upm.edu.my (I.R.); jafar@upm.edu.my (J.A.); azahy@upm.edu.my (N.A.Y.); 2 Institute of Advanced Technology, Universiti Putra Malaysia, UPM Serdang 43400, Selangor, Malaysia; amir1348@gmail.com; 3 inLAZER Dynamics Sdn Bhd, InnoHub Unit, Putra Science Park, Universiti Putra Malaysia, UPM Serdang 43400, Selangor, Malaysia; yasminmustaphakamil@gmail.com; 4 Wireless and Photonics Network Research Centre, Faculty of Engineering, Universiti Putra Malaysia, UPM Serdang 43400, Selangor, Malaysia; mam@upm.edu.my

**Keywords:** polyamidoamine dendrimer, reduced graphene oxide, dengue virus

## Abstract

This paper proposes a novel idea to enhance the sensitivity and selectivity of surface plasmon resonance (SPR) optical sensor for detection of dengue virus type-2 envelope proteins (DENV-2 E-proteins) using polyamidoamine (PAMAM) dendrimer biopolymer-based nanocomposite thin film. For this purpose, two ranges of DENV-2 E-protein concentrations, i.e., 0.000008–0.0001 nM and 0.00008–0.005 nM were evaluated, and the lowest detectable concentration was achieved at 0.00008 nM. The incorporation of PAMAM dendrimer-based nanocomposite thin film with an SPR sensor exhibited a significant increase in sensitivity and binding affinity to a lower range DENV-2 E-protein concentrations. Moreover, the proposed sensor displayed good selectivity towards DENV-2 E-proteins and have an average recovery of 80–120%. The findings of this study demonstrated that PAMAM dendrimer-based nanocomposite thin film combined with SPR sensor is a promising diagnostic tool for sensitive and selective detection of DENV-2 E-proteins.

## 1. Introduction

Surface plasmon resonance (SPR) has become an important optical sensing technique in the field of biomedical analysis, food safety, and chemistry because of their high sensitivity, fast analysis speed, real time, and label free detection [1,2,3,4,5]. In general, SPR is a direct-reading detection method that monitors the changes in refractive index on the gold surface when the surface plasmon is excited by the evanescent field under total internal reflection condition. Such resonance takes place at a certain incident angle with the formation of a minimum dip of reflected light intensity. This dip location is a robust function of the medium’s refractive index close to the interface, hence its potential use as a sensitive refractive index sensor [6,7,8,9,10,11,12]. However, the sensitivity of gold film-based SPR sensor is limited to the detection of any solutions that have a similar refractive index as the mass change on the binding is not sufficient to cause a detectable change in refractive index [13,14,15,16]. To overcome this limitation, a gold film surface was functionalized with a high density of biomolecules [17,18,19,20,21,22,23,24,25].

Engineering the SPR-gold film with high surface area materials are beneficial for enhancing the sensitivity of SPR sensors. Polyamidoamine (PAMAM) dendrimers are one of the smallest and most precise biopolymers available today since they were commercially available and, more importantly, could easily act as bio-conjugating reagents to increase the active sites of the sensor [26,27,28]. They are hydrophilic and are a family of highly branched macromolecules with different active functional groups outside its surface and so have advantages over other micromolecules due to monodispersity, modifiable surface functionalities, and high mechanical and chemical stability [29,30,31,32]. There is a demand for enhancing the adsorption of biomolecules (such as dengue proteins), and amine-reduced graphene oxide (NH_2_rGO) nanocomposites are beneficial to bind directly to a globular-shaped polyamidoamine (PAMAM) dendrimer without affecting their biological activity. In particular, many authors have demonstrated the capability of rGO as a sensing film because of its unique physiochemical properties including good reaction yields, excellent stability, reliable preparation, large surface areas, and good biocompatibility [33,34,35,36,37,38,39,40,41,42]. This NH_2_rGO–PAMAM composite is believed to have a great sensing performance in detecting and quantifying the dengue virus.

Dengue virus (DENV) is a mosquito-borne viral disease, comprising of four serotypes of the virus, i.e., DENV-1, DENV-2, DENV-3, and DENV-4 [43,44,45]. Infections with DENV often appear as non-specific symptoms such as mild fever, headache, and body rashes, and further delayed diagnosis might lead to dengue hemorrhagic fever, dengue shock syndrome, or death [46,47,48,49,50]. In this case, optical diagnostic has been vastly utilized in the relevance to dengue disease compared to the laboratory serological and commercial kits [51,52,53]. This is because the optical diagnostic, mainly for SPR sensor, focus on the measurement of a change in the optical properties of the sensor surface due to the adsorption of analyte. Most works on DENV detection using SPR sensor were listed in Table 1 [54,55,56,57,58,59,60,61,62,63].

It can be observed that the most targeted determinants were specific DENV antibodies and NS1 virus/proteins. Despite the success of DENV detection by using SPR, both determinants have some limitations in providing the early detection of DENV. The antibodies are released in response to DENV up to 7 days post-infection, while NS1 products are produced within 5 days of infection after cleavage from E-proteins. Additionally, detection of antibodies (IgM and IgG) is not always highly specific to DENV as it can be cross-reactive against other flavivirus, while detection of NS1 tends to be less sensitive in secondary dengue (DENV-2) infection than in primary dengue (DENV-1) infection [64,65,66,67]. To overcome these issues, the envelope (E) proteins of the DENV-2 has become our determinant. The E-proteins are the protein structures that form the coat of the host–virus itself, therefore it is enough to mount sufficient immune response earlier (in the viremia phase).

In this work, we propose to develop polyamidoamine dendrimer biopolymer-based nanocomposite thin film to improve the performance of SPR sensor for detection of dengue envelope proteins in terms of their sensitivity, binding affinity, and selectivity. Despite this interest, no other study to the best of our knowledge has achieved lowest detectable concentration DENV-2 E-proteins at 0.00008 nM (0.08 pM). The stability and spike recovery of the proposed SPR sensor was also evaluated in this work.

## 2. Materials and Methods

### 2.1. Reagents

Dithiobis(succinimidyl undecanoate) (DSU, >90% Dojindo, Japan), graphene oxide (Graphanea, Gipuzkoa, Spain), Polyamidoamine dendrimer (PAMAM, ethylenediamine, generation 4.0, 10 weight percent (wt. %) in methanol, Sigma Aldrich, St. Louis, MO, USA), Ethylenediamine (EDA, >99%, Sigma Aldrich, St. Louis, MO, USA), N-hydroxysuccinimide (NHS, 98%, Sigma Aldrich, St. Louis, MO, USA), 1-Ethyl-3-(3-(dimethylaminopropyl) carbodiimide hydrochloride (EDC, Thermo Fisher Scientific, Waltham, MA, USA), recombinant dengue virus type 2 envelope protein (DENV-2 E-proteins, Meridian Life Science, Inc., Tennessee, United States), specific monoclonal antibodies dengue virus type 2 envelope protein (IgM, Meridian Life Science, Inc., Memphis, TN, USA), recombinant dengue virus type 1 envelope protein (DENV-1 E-proteins, Meridian Life Science, Inc., Memphis, TN, USA), and recombinant zika envelope proteins (ZIKV, Mybiosource, San Diego, CA, USA) were used without further purification.

### 2.2. Preparation of NH_2_rGO–PAMAM-Based Nanocomposite Thin Film

Preparation of NH_2_rGO was begun by amalgamating the graphene oxide with EDC for 5 min and was later added by EDA. The mixture was then stirred vigorously with the aid of the magnetic stirrer to dissolve the suspension until it turns to dark black. The suspension was washed with ethanol and centrifuged immediately at high speed to discard the excess EDA and EDC. Then, the obtained NH_2_rGO was dried at 60 °C for at least 1 h. After the drying process, the resulting product was mixed with PAMAM solution. Unless otherwise stated, all the antibodies and antigen solutions were diluted in 10 mM phosphate buffered saline (PBS) at pH 7.4. All chemicals used in this work were of reagents or higher grade.

**Figure 1:**
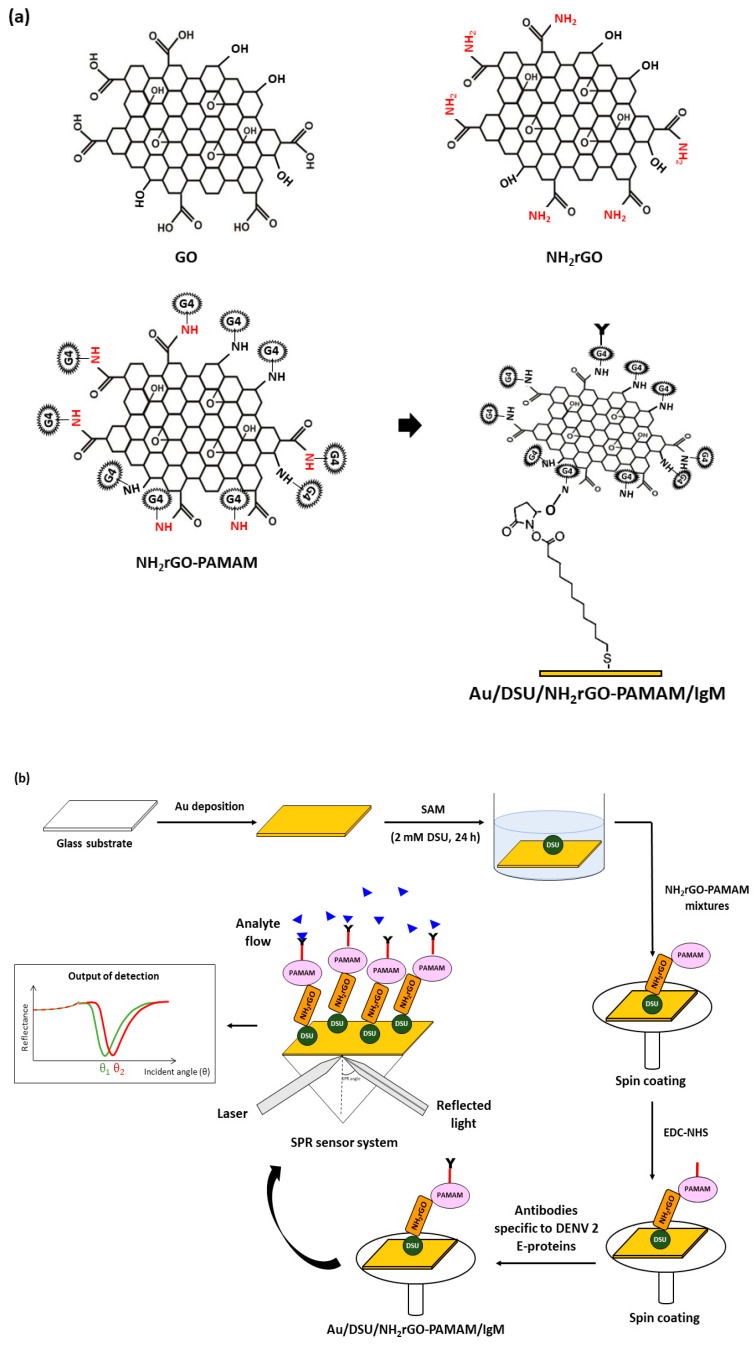
Illustration of (**a**) possible mechanism for the preparation of DSU/NH_2_rGO–PAMAM/IgM and (**b**) sensor film preparation.

A cleaned glass film (Menzel glass, 2.4 cm × 2.4 cm) was sputtered with a thin gold layer of 48 nm thick as a sensitive element of the SPR sensor using SC7640 Sputter Coater (I = 20 mA). To generate self-assembled monolayer (SAM), a gold-coated film was rinsed with water and ethanol followed by drying under nitrogen flow. The dimethyl sulfoxide solution containing 2 mM dithiobis (succinimidyl undecanoate) was prepared for adsorptions of thiol and disulphide onto substrate film. After 24 h, the SAM was formed and then thoroughly rinsed with acetone and subsequently with phosphate-buffered saline (PBS, pH 7.4). Briefly, an exact amount of 0.5 mL of the NH_2_rGO–PAMAM composite solution was dropped onto the substrate surface followed by spinning for 30 s. Thereafter, a substrate was incubated in EDC/NHS solution for 30 min and was later subjected to spinning process. After cross-linking, the substrate was immobilized with specific DENV antibodies (0.01 µM in PBS) to detect DENV-2 E-proteins selectively. The design of Au/DSU/NH_2_rGO–PAMAM/IgM sensor film is shown in Figure 1.

### 2.3. Incorporation of Au/DSU/NH_2_rGO–PAMAM/IgM Sensor Film into SPR System

The SPR measurement was then performed using a custom-built SPR system under Kretschmann configuration, which consisted of a helium-neon laser, an optical stage features an angular resolution of 0.001° with a stepper motor driven version (Newport MM 3000), a polarizer, an optical chopper (SR 540), and a prism. In this system, PBS solution was used to execute the baseline data. Approximately, 100 µL of diluted DENV-2 E-proteins was subsequently injected into the o-ring. All experiments were repeated three times with a new sensor film for each concentration of DENV-2 E-proteins. The increment/decrement of SPR reflectance was calculated by taking the difference between the SPR reflectance curves of the PBS solution and the respective analyte solution, DENV-2 E-proteins.

## 3. Results

### 3.1. FTIR Analysis of Au/DSU/NH_2_rGO–PAMAM/IgM Sensor Film

**Figure 2:**
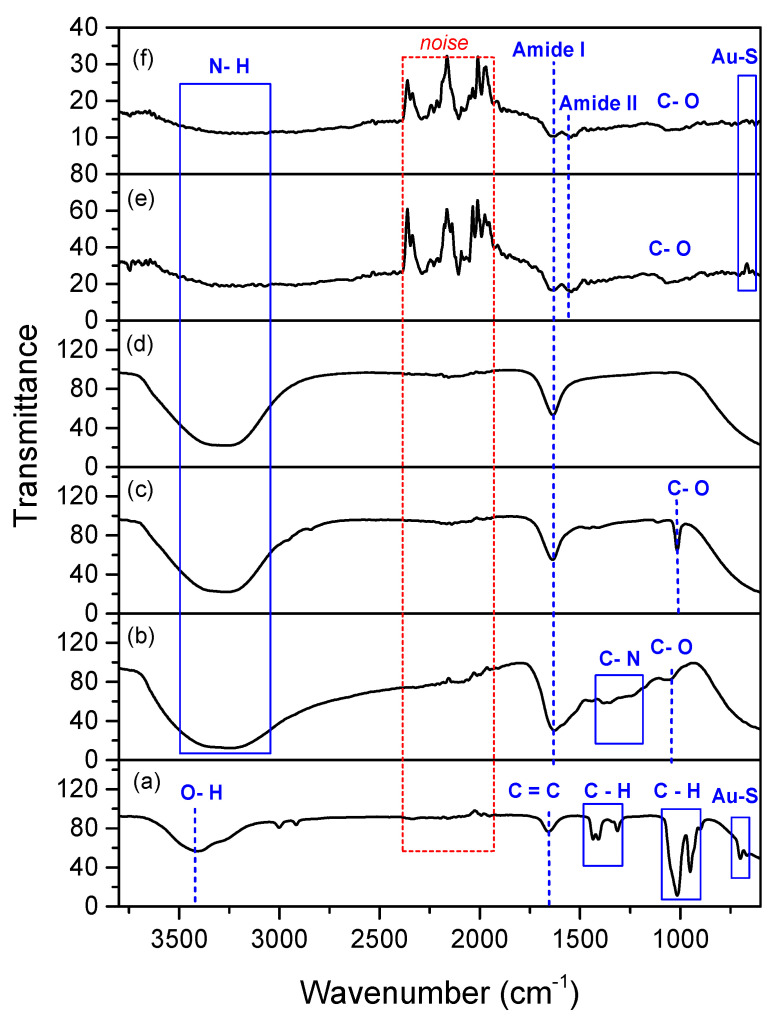
FTIR spectra of (**a**) DSU; (**b**) NH_2_rGO; (**c**) PAMAM; (**d**) IgM; (**e**) Au/DSU/NH_2_rGO-PAMAM/IgM sensor film; (**f**) Au/DSU/NH_2_rGO-PAMAM/IgM sensor film exposed to DENV-2 E-proteins.

FTIR spectra of DSU, NH_2_rGO, PAMAM, and IgM was conducted using FTIR spectroscopy (VERTEX 70) to confirm the development of Au/DSU/NH_2_rGO–PAMAM/IgM sensor film (Figure 2). Four characteristic peaks of DSU positioned at 3420 cm^−1^, 1656 cm^−1^, 1424–1300 cm^−1^, 1017–940 cm^−1^, and 650–690 cm^−1^ can be attributed to the O-H stretching, C=C stretching, C-H bending, C-H bending, and Au-S band, respectively. In the spectrum of NH_2_rGO, there are bands from N-H stretching (3200–3346 cm^−1^), Amide I (1600 cm^−1^), C-N stretching (1250–1344 cm^−1^), and C-O stretching (1051 cm^−1^) [68]. For PAMAM spectrum, the obvious three peaks were attributed to the N-H stretching, Amide I, and C-O stretching [69,70], while two absorption peaks in IgM spectrum was assigned to the N-H stretching and Amide I [71,72]. As seen from the spectrum of Au/DSU/NH_2_rGO–PAMAM/IgM sensor film, there are the absorption bands due to the N-H stretching, Amide I, Amide II, C-O stretching, and Au-S band. The successful of IgM immobilization can be confirmed by a reduction of N-H band and the appearance of a small peak of Amide II. Upon introduction of DENV into the sensor film, the peaks for N-H stretching and Amides faced an intensity reduction, proving the immunological reaction between the antibodies and DENV-2 E-proteins [73].

### 3.2. SPR Analysis of Au/DSU/NH_2_rGO–PAMAM/IgM Sensor Film towards DENV-2 E-Proteins

#### 3.2.1. SPR Reflectivity

**Figure 3:**
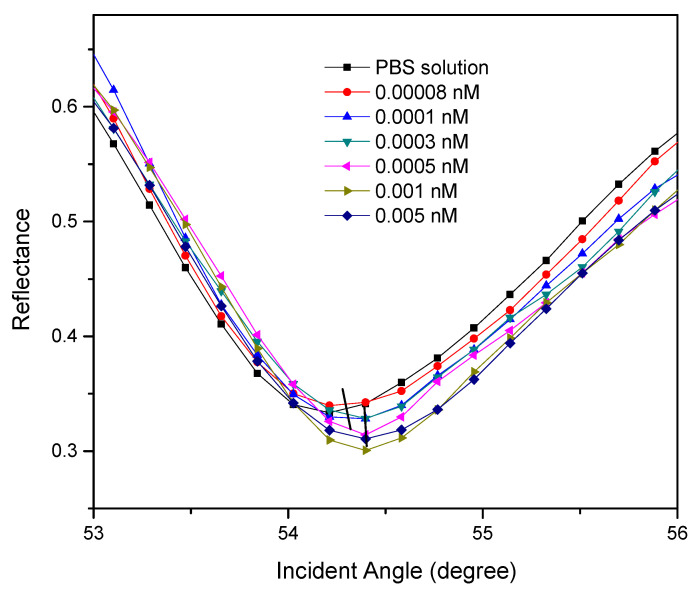
The reflectance curves of 0.00008–0.005 nM of DENV-2 E-protein detection.

Figure 3 shows the SPR reflectance curves for Au/DSU/NH_2_rGO–PAMAM thin film with high-range DENV-2 E-protein concentrations of 0.00008–0.005 nM. Prior to the analyte detection, the PBS solution was injected into the o-ring, producing a resonance angle of 54.213°. The analyte detection was then carried out by injecting the high-range DENV-2 E-protein concentrations of 0.00008–0.005 nM one after another into the o-ring. The first obtained resonance angle of 0.00008 nM of DENV-2 E-proteins was 54.305°. Next, the Au/NH_2_rGO–PAMAM thin film showed an increase in resonance angles of the incident light towards 54.313°, 54.392°, 54.400°, and 54.408° due to the introduction of DENV-2 E-protein concentrations of 0.0001 nM, 0.0003 nM, 0.0005 nM, and 0.001 nM, respectively. The increment of resonance angles can be interpreted as the increment of antigens attached to the sensor surfaces. It was found that the rise in resonance angle shifts of all DENV-2 E-protein concentrations, i.e., Δθ_SPR_ = 0.092°, 0.100°, 0.179°, 0.187°, and 0.195°, can be associated with the changes in the real part of the refractive index of the sensor surface caused by the binding of DENV-2 E-proteins, which consequently affect the thickness of the sensing layer [74,75]. When the introduction of DENV-2 E-proteins was higher than 0.001 nM, the reflectance curves remain unchanged at 54.408° due to the maximum binding of DENV-2 E-proteins. The observion of this phenomenon is mainly due to the difficulty of SPR evanescent wave in penetrating the thick dielectric layer, which then reduces the sensitivity of the SPR sensor [76,77].

**Figure 4:**
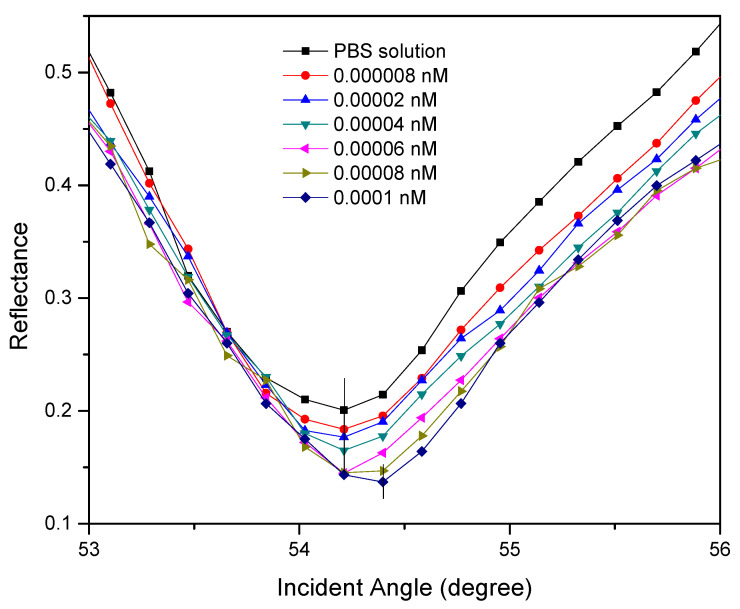
The reflectance curves of 0.000008–0.0001 nM of DENV-2 E-protein detection.

Next, the Au/DSU/NH_2_rGO–PAMAM/IgM-based SPR sensor response towards a lower range of DENV-2 E-protein concentration (0.000008 nM to 0.0001 nM) was conducted to determine the lowest detectable concentration for DENV-2 E-proteins, or on the other hand, to determine the detection limit obtained by this sensor. In this regard, the limit of detection (LOD) is derived by the capability of the sensor to distinguish the SPR response of DENV-2 E-protein detection and reference solution detection [78]. The results depicted in Figure 4 show that the resonance angles for 0.000008–0.00006 nM of DENV-2 E-proteins remain unchanged from the resonance angle of PBS solution, 54.211°. This is owing to the weak interaction of the low refractive index of DENV-2 E-proteins solution and the sensor layer, which cannot significantly increase the refractive index of the sensing layer. However, with increasing of DENV-2 E-protein concentrations, i.e., 0.00008 nM and 0.0001 nM, the resonance angles shifted to the higher angles of 54.311° and 54.393°, respectively. The increase in the resonance angle is strongly evidenced by the successful detection of DENV-2 E-proteins on the sensor surface, resulting in an increase in the refractive index near a gold layer [79,80]. It can be hypothesized that the lower limit of detection of this study is 0.00008 nM as any concentration less than 0.00008 nM is not detectable. The detection limit obtained was then compared with some of the recently published data as shown in Table 2, which clearly shows that the Au/DSU/NH_2_rGO–PAMAM/IgM sensor film-based SPR sensor has the lowest detection limit so far [62,63,73,81,82]. Herein, the inclusion of DSU as a self-assembly monolayer and NH_2_rGO–PAMAM composite as a sensing layer has provided strong support for IgM immobilization for selective detection of dengue virus.

#### 3.2.2. SPR Performances

**Figure 5:**
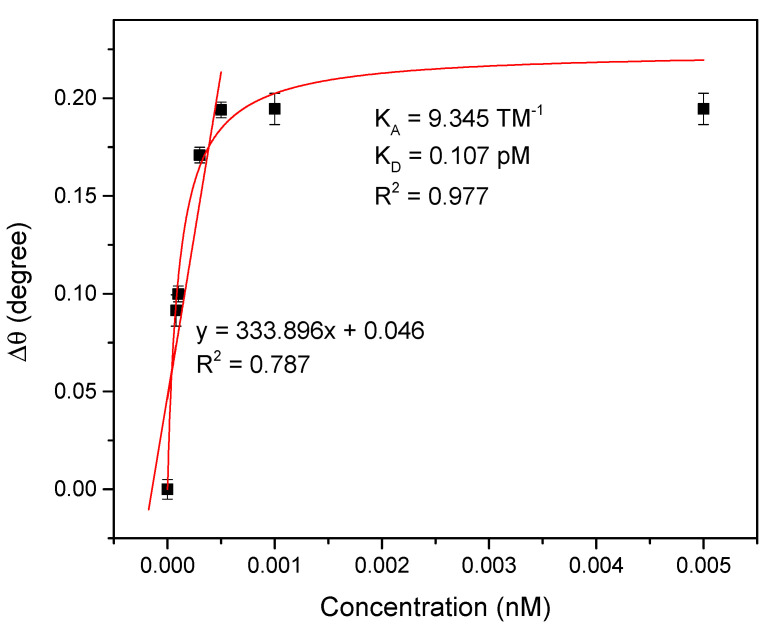
A linear regression graph and Langmuir graph for Au/DSU/NH_2_rGO–PAMAM/IgM sensor film.

Figure 5 shows a linear regression graph of the shift in SPR angle versus DENV-2 E-protein concentrations ranging from 0.00008 to 0.0005 nM. The gradient of the linear fit was 333.896 °/nM with the R^2^, of 0.787 (standard deviation ±0.02). Based on the gradient value, it was concluded that the sensitivity of Au/DSU/NH_2_rGO–PAMAM/IgM sensor film when detecting DENV-2 E-proteins was 333.896 °/nM. The results clearly indicated that the proposed sensor can be potentially used to detect the lowest concentration of DENV-2 E-proteins with a high sensitivity. This behavior can be understood because of the stronger penetration depth of the SPR evanescent field along the Au/DSU/NH_2_rGO–PAMAM/IgM sensor film, thus, it can significantly detect the DENV-2 E-proteins as low as 0.00008 nM using SPR technique [83,84,85]. The association constant, K_A_ and dissociation constant, K_D_ for the assessment of the interaction affinity were then calculated and found to be 9.345 TM^−1^ and 0.107 pM, respectively, with the R^2^ of 0.977. The smaller K_D_ value revealed that the Au/DSU/NH_2_rGO–PAMAM/IgM sensor film has a high affinity interaction with the DENV-2 E-proteins and is found to be consistent with the standard K_D_ value for protein interaction (K_D_ < 10 nM) [86,87].

**Figure 6:**
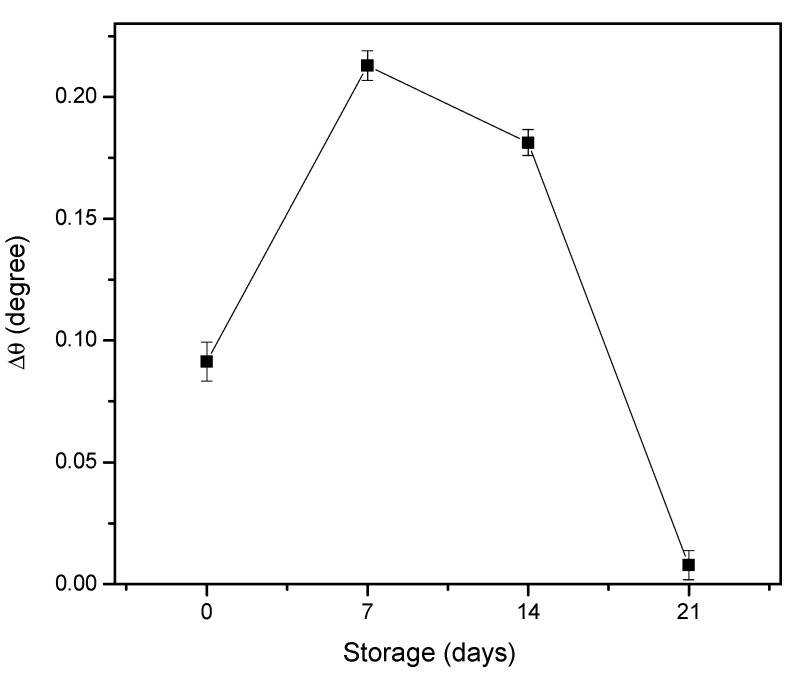
The stability test of Au/DSU/NH_2_rGO–PAMAM/IgM sensor film.

The proposed sensor films were then stored in a refrigerator for three weeks to examine the stability of the sensor for 0.08 pM of DENV-2 E-protein detection. Figure 6 shows that the resonance angle decreased dramatically on the 21st day storage. However, the Au/DSU/NH_2_rGO–PAMAM/IgM sensor film still provided a good resonance angle shift throughout 7-day storage. This result suggested that the antibodies immobilization on the sensor surface is strongly retained without losing their bonding.

**Figure 7:**
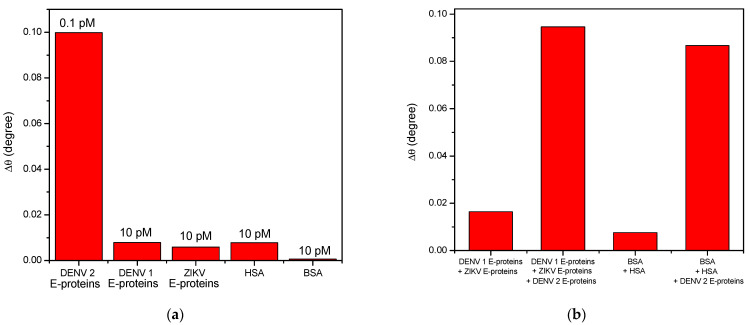
Selectivity test between Au/DSU/NH_2_rGO–PAMAM/IgM sensor film and (**a**) each different analyte; (**b**) mixture of analytes.

To evaluate the selectivity performance of the proposed sensor, other 10 pM competitor analytes, i.e., DENV-1 E-proteins, ZIKV E-proteins, HSA, and BSA, were selected and tested against 0.1 pM DENV-2 E-proteins as shown in Figure 7a. As can be observed, the introduction of HSA proteins and DENV-1 E-proteins resulted in a rise in SPR responses, which might be due to the non-specific interactions between proteins and sensor surface. As expected, the binding between DENV-1 E-proteins and sensor surface occurred because of 65% of single-stranded RNA genomes were shared by each serotype of DENV [88,89]. Meanwhile, a high SPR response for HSA proteins can be accounted for the excessive proteins in the blood with a molecular weight of 66.4 kDa when compared to 50 kDa DENV-1 E-proteins [90,91]. Due to a direct binding between DENV-2 E-proteins and its specific antibodies immobilized on the sensor surface, the SPR response of 0.1 pM of DENV-2 E-protein solution was obviously increased. It is verified that the antibodies immobilized on a sensor surface have a highly selective in detecting DENV-2 E-proteins.

Figure 7b depicts the selective shift of SPR angle of DENV-2 E-proteins in multiple analytes solution. The concentrations of each analyte were fixed at 10 pM. As expected, a markedly large shift of SPR angle was generated for multiple solutions containing DENV-2 E-proteins compared to other solution that does not have DENV-2 E-proteins. The results suggest that the proposed sensor exhibits excellent selectivity of the proposed sensor towards the sensing of DENV-2 E-proteins. This is due to the stronger binding of a very specific immobilized IgM at NH_2_rGO–PAMAM sensor layer.

**Figure 8:**
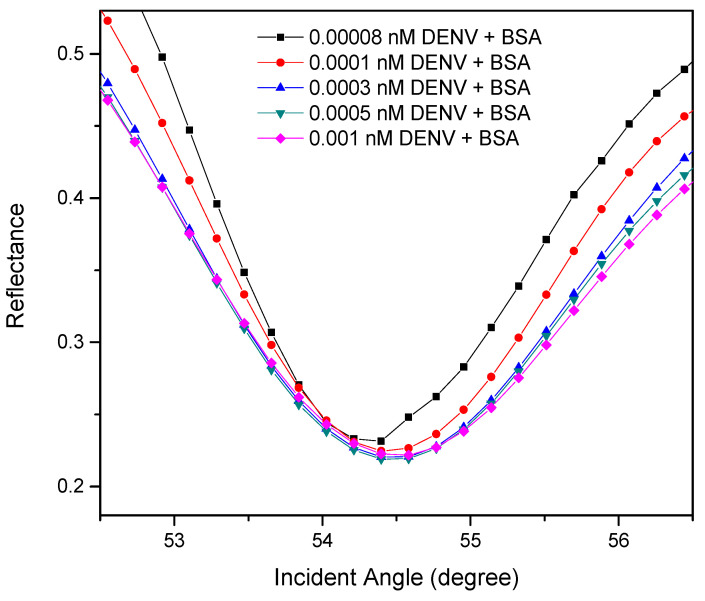
Experimental SPR curves for different concentration of DENV-2 E-proteins in spiked samples.

To further validate the applicability of the proposed sensor as DENV immunosensor in real samples, 10% BSA solutions were spiked into all concentrations of DENV-2 E-proteins under the same procedures. According to the SPR response in Figure 8, the recoveries of DENV-2 E-proteins was calculated and tabulated in Table 3. The obtained average recovery was consistent with acceptable recovery, which is in the range of 80–120%, indicates that the proposed sensor can be used for detection and quantification of DENV-2 E-proteins in real samples [92,93,94,95].

## 4. Conclusions

In this study, a highly sensitive and selective Au/DSU/NH_2_rGO–PAMAM/IgM thin-film-based SPR sensor was successfully developed for detection of DENV-2 E-proteins of 0.000008–0.005 nM. The SPR results show that the proposed sensor successfully quantifies the concentration of targeted DENV-2 E-proteins as low as 0.00008 nM with a sensitivity value of 333.896 °/nM. The proposed sensor film also showed a strong binding affinity constant of 9.345 TM^−1^, good stability within 7-day storage, and a good selective response towards DENV-2 E-proteins. Furthermore, the obtained average recovery was in the acceptable range of 80–120%, demonstrating that this novel approach could provide a fast sensor platform option for the future of dengue diagnostics.

## Figures and Tables

**Table 1:** Dengue virus (DENV) detection using surface plasmon resonance (SPR) sensor.

Targeted Determinant	Detection Limit	References
IgM	-	[54,55,56,57]
DENV	-	[58]
DENV-2 NS1	0.25 ng/mL	[59]
NS1 proteins	1 nM	[60]
NS1	5.73 pg/mm^2^	[61]
IgM	2.125 pM	[62]
NS1 proteins	0.3 nM	[63]

**Table 2:** Recent studies and comparison of detection limit for DENV based on optical sensor.

Optical Sensor	Active Layer	Determinant	Detection Limit	References
Optical fiber	PAMAM ^1^	DNEV-2 E-proteins	1 pM	[73]
Optical fiber	-	DENV NS1 IgG antibody	200 pM	[81]
SPR	CM5/EDC-NHS ^2^	IgM antibody	2.125 pM	[62]
SPR	CMD/EDC-NHS ^3^	NS1 proteins	0.3 nM	[63]
Colorimetric	G4-hemin DNAzyme	DENV-1 DNA; DENV-2 DNA; DENV-3 DNA, DENV-4 DNA	8.8 nM; 4.9 nM; 9.3 nM; 5.1 nM	[82]
SPR	Au/DSU/NH_2_rGO–PAMAM	DENV-2 E-proteins	0.08 pM	This work

^1^ Polyamidoamine dendrimer, ^2^ carboxymethyl dextran matrix/N-ethyl-N-(dimethylaminopropyl) carbodiimide-N-hydroxysuccinimide, ^3^ carboxyl methildextrand/N-ethyl-N-(dimethylaminopropyl) carbodiimide-N-hydroxysuccinimide.

**Table 3:** Spike and recovery results of DENV-2 E-proteins in BSA.

Sample Concentration (pM)	Spike (%)	PBS + DENV-2 E-Protein Resonance Angle (Degree)	BSA + DENV-2 E-Protein Resonance Angle (Degree)	Recovery (%)
0.08	4.5	54.305	54.400	100.174
0.1	4.5	54.313	54.480	100.307
0.3	4.5	54.392	54.496	100.191
0.5	4.5	54.400	54.496	100.176
1	4.5	54.408	54.500	100.169

## Data Availability

Data sharing not applicable.
